# Neck circumference as an independent indicator to non-alcoholic fatty liver disease in non-obese men

**DOI:** 10.1186/s12986-015-0060-z

**Published:** 2015-12-30

**Authors:** Qin Li, Ningjian Wang, Bing Han, Yi Chen, Chunfang Zhu, Yingchao Chen, Fangzhen Xia, Zhen Cang, Chaoxia Zhu, Chi Chen, Hualing Zhai, Boren Jiang, Dongping Lin, Yingli Lu

**Affiliations:** Institute and Department of Endocrinology and Metabolism, Shanghai Ninth People’s Hospital, Shanghai JiaoTong University, School of Medicine, No.639 Zhizaoju Road, Shanghai, 200011 China

**Keywords:** Neck circumference, NAFLD, Non-obese

## Abstract

**Background:**

To investigate the relationship of the neck circumference (NC) with non-alcoholic fatty liver disease (NAFLD) in non-obese Chinese population.

**Methods:**

Our data were obtained from a cross-sectional survey on the prevalence of metabolic diseases and risk factors in East China in 2014. Subjects with a BMI ≥ 18.5 kg/m^2^ and < 25 kg/m^2^ were considered normal weight. A total of 2668 participants aged 18–89 were identified for analysis. Anthropometric indices, biochemical parameters, clinical characteristics and abdominal ultrasound were measured. Independent predictors of NAFLD were identified by multiple logistic regressions.

**Results:**

The overall prevalence of NAFLD was 10.94 % in this study population and men had a higher prevalence than women (19.89 % vs 7.48 %, *P <* 0.01). The mean NC was greater in NAFLD subjects compared with other groups in both genders (*P <* 0.01). NC was correlated to BMI, waist circumference, hip circumference, systolic blood pressure, diastolic blood pressure, insulin, HOMA-IR, triglycerides and ALT, regardless of sex. In the highest quartile of NC levels in men but not in women, the risks were substantially higher for NAFLD [odds ratio 2.18, (95 % confidence interval 1.16–4.13)] (*P <* 0.001 for trend) after adjusting for potential confounding factors.

**Conclusion:**

NC was an independent indicator for NAFLD in normal weighted men.

## Background

The distribution of body fat is an important factor that determines metabolic health [1]. Many researchers have shown that upper-body subcutaneous adipose tissue may confer additional risk for metabolic disorders beyond overall and central adiposity [2, 3]. The neck circumference (NC) measurement is a surrogate marker of determining upper-body subcutaneous distribution and the close correlations between NC and various metabolic risk factors have been widely explored [3–5].

Non-alcoholic fatty liver disease (NAFLD) has a close relationship with components of metabolic syndrome and is considered the hepatic manifestation of metabolic syndrome [6]. Although NAFLD is the most common cause of abnormal liver function tests owing to the rapid rise in the prevalence of obesity, now the high prevalence of NAFLD in normal weight people has drawn great attention[7]. NAFLD is usually considered an incidental pathologic finding, with scarce or no clinical symptoms. Hence, identifying NAFLD in those with a normal weight might have important clinical significance. Early diagnosis and management would also effectively improve its prognosis [8, 9].

From a clinical perspective, NAFLD is characterized by ectopic fat deposition [10]. NC is an alternative measurement for upper-body subcutaneous fat and is also a good indicator of ectopic fat distribution [4, 11]. Therefore, NC measurement may play a vital role in NAFLD clinical prediction. Although NC now has been proposed as an effective predictor for NAFLD in the general population [8], no detailed data is available to describe the relationship between the NC and NAFLD in normal weight people. Here we conduct this study to obtain a better understanding of the associations between NC and NAFLD and other metabolic risk factors.

## Methods

### Subjects

A cross-sectional survey to evaluate the prevalence of metabolic diseases and risk factors in East China was performed in 2014 (SPECT-China, ChiCTRECS-14005052, www.chictr.org). The details of the study design have been described previously [12]. In brief, the study was conducted from February to June 2014. Six residential areas in Shanghai, seven in Jiangxi province and three in Zhejiang province were selected using a stratified and cluster sampling method. Using included and excluded criteria as presented before, a total of 7200 Chinese residents participated in this investigation. After exclusion of participants who had complete missing laboratory results (*n =* 183), missing questionnaire data (*n =* 112) and were younger than 18-year-old (*n =* 6), 6899 subjects were enrolled in SPECT-China study finally [12]. The study design and procedures were approved by the Ethics Committee of Shanghai Jiaotong University Affiliated Ninth People’s Hospital, and written informed consents were obtained from all participants.

**Fig. 1:**
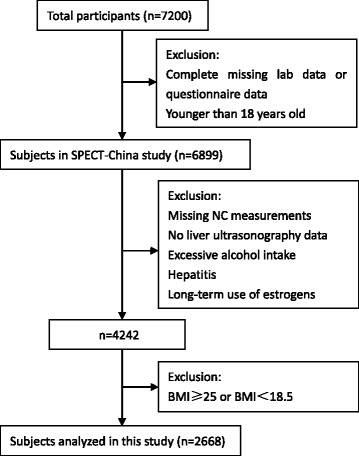
Flowchart of participants’ inclusion and exclusion

Normal weight was defined as BMI ≥ 18.5 kg/m^2^ and BMI < 25 kg/m^2^. Candidates were excluded according to the following additional criteria: previous/current excessive alcohol intake (male > 20 g/d, female > 10 g/d), hepatitis, long-term use of estrogens, goiter or other neck masses or deformity with ultrasound. Finally, 2668 individuals aged 18–89 years were analyzed. The participants’ inclusion and exclusion in this analysis were show in Fig. 1.

### Anthropometric and laboratory evaluation

Data collection was conducted by trained staff according to a standard protocol. A questionnaire including information on demographic characteristics, medical history, and lifestyle factors was administered by trained interviewers [13]. Anthropometric measurements were taken to obtain height and weight according to standard technique by trained investigators. Participants stood on the weight scale (body-weight balance attached with height gauge) with bare feet close together, arms at the side and wore little clothing [14]. Body mass index (BMI) was calculated as the weight in kilograms divided by the square of the height in meters. Waist circumference (WC) was measured on the midaxillary line between the lower border of the rib cage and the upper margin of the iliac crest. NC was measured with head erect and eyes facing forward, horizontally at the upper margin of the laryngeal prominence with a flexible tape [2]. Insulin resistance was estimated by the homeostatic model assessment (HOMA-IR) index: [fasting insulin (mIU/L)] *[FPG (mmol/L)]/22.5. Modification of diet in renal disease (MDRD) equation recalibrated for Chinese was used to estimate eGFR expressed in ml/min/ 1.73 m^2^: eGFR = 186 * [SCR *0.011]^1.154^ *[age]^0.203^ * [0.742 if female] *1.233, where SCR is serum creatinine expressed as mmol/l and 1.233 is the coefficient for Chinese [15].

Peripheral venous blood samples were collected after an overnight fast of at least 8 h. All samples were centrifuged immediately after collection and then shipped by air in dry ice to a central laboratory within 2–4 h of collection. Glycated hemoglobin (HbA1c) was assessed by high-performance liquid chromatography (MQ-2000PT, China). Plasma glucose, alanine aminotransferase (ALT), aspartate aminotransferase (AST) and lipid profile including total cholesterol (TC), triglycerides (TG), high density lipoprotein-cholesterol (HDL-c) and low density lipoprotein-cholesterol (LDL-c) were measured by BECKMAN COULTER AU 680 (Germany). Insulin was detected by chemiluminescence method (Abbott i2000 SR, USA).

### NAFLD evaluation

Guidelines for the diagnosis of NAFLD proposed by the Asia–Pacific Working Party were used [15, 16]. NAFLD was clinically defined as manifestations of B ultrasonography, ruling out the habit of drinking and the history of specific diseases that could result in fatty liver [15]. Abdominal ultrasonography was performed by experienced radiologists who were blinded to clinical presentation and laboratory findings [15]. Hepatic steatosis was defined as a diffuse increase of fine echoes in the liver parenchyma compared with that in the kidney or spleen parenchyma based on standard criteria [15].

### Statistical analysis

Data management and statistical analyses were performed using IBM SPSS Statistics, Version 22 (IBM Corporation, Armonk, NY, USA). Normally distributed data were expressed as mean ± SD, whereas variables with a non-normally distribution were reported as median with interquartile range and were logarithmically transformed before analysis. Comparisons between groups were tested by Student’s t-test and analysis of variance (ANOVA), in case of normally distributed variables; and Mann–Whitney U and Kruskal-Wallis test for skewed distributed variables. The composition of rate was calculated by Chi-square tests. Spearman’s correlation coefficient was employed to test the correlations between different variables. To identify independent risk factors of NAFLD, multiple logistic regressions models were used and variables that were biologically likely to be related with NAFLD were analyzed. Odds ratio (OR) per standard deviation was used to show the relative strength of the relationship. Two-sided p values <0.05 were considered significant.

## Results

### Patient characteristics

Of the 2668 subjects evaluated, mean age was 50.07 ± 14.09 years with a slight female preponderance (F: M = 2.6:1), for men were more likely to be obesity. Overall, the prevalence of NAFLD was 10.94 % in this study population, and was 19.89 % in men and 7.48 % in women. Clinical characteristics of the participants were presented in Table 1, separately according to the severity of liver fat infiltration. In men, age changed minimally with NAFLD status. While in women, mean age levels gradually increased from normal people to NAFLD subjects (*P <* 0.001), which was in agreement with previous studies both in normal weighted people and in the general population. NC, along with other anthropometric measures such as body weight, WC, hip circumference (HC) and BMI, tended to be higher with greater NAFLD severity in both genders. The blood pressure, fasting blood glucose, fasting insulin, HbA1c, HOMA-IR, TC, TG, and LDL-c, as well as ALT, AST, showed a graded increase with increasing severity of NAFLD in women (*P <* 0.01). But in males, such tendencies were only observed in fasting insulin, HOMA-IR, TG and LDL-c (*P <* 0.05). The mean HDL-c and eGFR of the participants were the lowest in the NAFLD group, followed by mild group and normal group in both genders (all *P <* 0.001).

**Table 1:** General characteristics of participants categorized severity of liver fat infiltration

	Men				Women			
	Normal	Mild	Moderate to severe	*P*	Normal	Mild	Moderate to severe	*P*
*N*	435	161	148		1470	310	144	
Age (yr)	52.03 ± 15.06	47.81 ± 15.88	49.16 ± 15.56	0.06	48.24 ± 13.80	54.55 ± 12.24	56.58 ± 10.71	<0.01
Weight (kg)	61.15 ± 6.42	65.03 ± 6.52	66.29 ± 6.69	<0.01	54.10 ± 5.42	55.95 ± 5.44	57.44 ± 4.45	<0.01
Neck circumference (cm)	34.08 ± 2.16	34.78 ± 2.04	35.45 ± 2.10	<0.01	30.92 ± 2.25	31.33 ± 1.96	31.99 ± 2.09	<0.01
Waist circumference (cm)	76.17 ± 6.60	78.43 ± 6.70	80.08 ± 6.27	<0.01	71.09 ± 6.85	75.08 ± 6.05	78.18 ± 6.68	<0.01
Hip circumference (cm)	89.56 ± 4.66	91.77 ± 5.60	92.16 ± 5.03	0.87	89.56 ± 4.73	90.39 ± 5.18	91.14 ± 4.92	<0.01
Waist to hip ratio	0.87 ± 0.07	0.86 ± 0.07	0.87 ± 0.47	0.04	0.80 ± 0.07	0.83 ± 0.07	0.86 ± 0.47	<0.01
BMI (kg/m^2^)	21.98 ± 1.69	22.46 ± 1.70	23.13 ± 1.46	<0.01	21.90 ± 1.69	22.84 ± 1.46	23.48 ± 1.27	<0.01
SBP (mmHg)	125.74 ± 19.27	127.64 ± 17.90	129.88 ± 17.83	0.06	121.98 ± 20.11	128.71 ± 19.59	133.57 ± 21.48	<0.01
DBP (mmHg)	76.69 ± 11.64	77.24 ± 12.90	78.23 ± 12.05	0.41	72.75 ± 11.69	77.01 ± 11.90	79.56 ± 12.75	<0.01
FBG (mmol/L)	5.42 ± 1.36	5.29 ± 0.99	5.53 ± 1.41	0.28	5.35 ± 0.93	5.62 ± 1.13	6.02 ± 1.51	<0.01
Fasting insulin (pmol/L)	25.6(17.8–33.3)	27.4(20.0–38.6)	31.6(22.2–46.4)	<0.01	30.1(22.6–40.6)	33.8(24.7–46.1)	40.9(29.6–56.5)	<0.01
HbA1c (%)	5.28 ± 0.88	5.24 ± 0.70	5.43 ± 1.10	0.14	5.12 ± 0.60	5.30 ± 0.73	5.48 ± 0.89	<0.01
HOMA-IR	0.85(0.57–1.17)	0.90(0.63–1.30)	1.06(0.73–1.65)	<0.01	1.03(0.73–1.39)	1.17(0.85–1.65)	1.57(1.12–2.16)	<0.01
Total cholerterol (mmol/L)	4.84 ± 0.92	4.70 ± 0.89	4.89 ± 1.17	0.21	4.89 ± 0.93	5.15 ± 1.09	5.26 ± 0.89	<0.01
LDL-cholesterol (mmol/L)	2.76 ± 0.66	2.82 ± 0.61	2.97 ± 0.70	<0.01	2.77 ± 0.69	2.94 ± 0.75	3.08 ± 0.62	<0.01
HDL-cholesterol (mmol/L)	1.40 ± 0.28	1.32 ± 0.27	1.28 ± 0.27	<0.01	1.57 ± 0.30	1.50 ± 0.31	1.40 ± 0.28	<0.01
Triglycerides (mmol/L)	1.10(0.87–1.48)	1.24(0.91–1.76)	1.50(1.02–2.05)	<0.01	1.03(0.77–1.38)	1.32(0.95–1.77)	1.77(1.34–2.45)	<0.01
Creatinine (umol/L)	87.01 ± 14.31	88.23 ± 12.04	90.39 ± 13.16	0.03	68.75 ± 11.39	68.61 ± 11.15	69.80 ± 10.33	0.53
eGFR (ml/min/1.73^2^)	91.58 ± 17.58	91.03 ± 14.85	87.82 ± 13.14	0.05	90.29 ± 16.63	88.22 ± 17.42	85.04 ± 14.05	<0.01
ALT(U/L)	19(15–25)	18(14–25.5)	22(16–32)	0.04	14(11–19)	16(13–21)	20(15–28.5)	<0.01
AST (U/L)	24(20–28)	22(19–27)	23(20–30)	<0.01	21(18–25)	22(19–26)	24(20.5–28)	<0.01
Smoker N, (%)	140(32.18 %)	53(32.92 %)	52(35.14 %)	0.04	20(1.36 %)	2(0.65 %)	3(2.1 %)	0.12

SBP: systolic blood pressure; DBP: diastolic blood pressure; FBG: fasting blood glucose

The characteristics of the study participants according to quartile of NC are presented in Table 2 and Table 3. The mean value of NC was 32.02 ± 2.68 cm in the total participants, and men had a NC 3.5 cm wider than women (34.50 ± 2.15 vs 31.03 ± 2.22 cm, *P <* 0.001). When analyzed by quartiles of NC levels, male individuals with higher NC had more extensive elevations in BMI, WC and systolic blood pressure, greater levels of fasting insulin, HOMA-IR and TG (all *P <* 0.001). The other potential risk factors analyzed, such as age, diastolic blood pressure, fasting blood glucose, HbA1c, TC level, LDL-c, HDL-c, ALT, AST and eGFR showed no graded changes as NC levels increased. At the mean time, female individuals with higher NC had more extensive elevations in BMI, WC and blood pressure, greater levels of fasting blood glucose, fasting insulin, HbA1c and HOMA-IR, higher levels of TC, TG, LDL-c, as well as ALT. In contrast, the participants with higher NC levels displayed lower levels of eGFR and HDL-c in women (all *P <* 0.001).

**Table 2:** Characteristics of study participants according to neck circumference quartiles in men

Men	Neck circumference (cm)				*P*
	≤33	33–34	34–36	>36	
*N*	229	156	246	113	
Age (yr)	52.55 ± 15.39	49.63 ± 15.50	49.39 ± 14.43	50.35 ± 14.14	0.11
weight (kg)	59.39 ± 7.75	62.44 ± 8.08	65.67 ± 8.16	66.18 ± 9.89	<0.01
Waist circumference (cm)	74.81 ± 7.91	76.82 ± 7.32	79.28 ± 7.07	81.46 ± 8.00	<0.01
Hip circumference (cm)	88.72 ± 5.68	89.44 ± 5.10	91.72 ± 5.65	93.78 ± 6.22	<0.01
Waist to hip ratio	0.84 ± 0.08	0.85 ± 0.06	0.90 ± 0.05	0.87 ± 0.07	0.24
BMI (kg/m^2^)	21.65 ± 2.52	22.16 ± 2.57	22.79 ± 2.50	23.43 ± 2.97	<0.01
SBP (mmHg)	124.05 ± 19.45	126.25 ± 19.17	127.47 ± 17.27	132.59 ± 17.01	<0.01
DBP (mmHg)	76.33 ± 12.67	76.32 ± 11.78	77.03 ± 12.09	78.06 ± 12.41	0.35
FBG (mmol/L)	5.40 ± 1.00	5.31 ± 1.56	5.37 ± 1.21	5.70 ± 1.67	0.08
Fasting insulin (pmol/L)	24.9(16.4–33.1)	27.2(19.2–39.9)	27.7(19.0–35.3)	30.4(21.2–45.8)	<0.01
HbA1c (%)	5.26 ± 0.67	5.25 ± 1.09	5.28 ± 0.86	5.47 ± 1.12	0.17
HOMA-IR	0.86(0.57–1.09)	0.88(0.56–1.39)	0.90(0.66–1.22)	1.05(0.72–1.61)	<0.01
Total cholerterol (mmol/L)	4.78 ± 0.84	4.80 ± 0.93	4.72 ± 0.82	5.14 ± 1.24	<0.01
LDL-cholesterol (mmol/L)	2.73 ± 0.60	2.80 ± 0.67	2.80 ± 0.59	3.09 ± 0.79	<0.01
HDL-cholesterol (mmol/L)	1.43 ± 0.30	1.36 ± 0.27	1.31 ± 0.25	1.34 ± 0.27	<0.01
Triglycerides (mmol/L)	1.10(0.81–1.37)	1.15(0.89–1.56)	1.25(0.90–1.80)	1.37(0.99–1.93)	<0.01
Creatinine (umol/L)	85.63 ± 13.87	88.08 ± 13.74	89.96 ± 15.26	88.86 ± 15.80	<0.01
eGFR (ml/min/1.73^2^)	93.11 ± 16.92	90.45 ± 16.56	89.03 ± 16.08	89.71 ± 16.57	0.04
ALT (U/L)	19(14–25)	21(15–29)	19(15–26)	19(15–29)	0.18
AST (U/L)	24(20–29)	24(20–29)	23(20–28)	22(19–27)	0.35
NAFLD, %	10.04 %	16.67 %	26.02 %	30.97 %	<0.01

**Table 3:** Characteristics of study participants according to neck circumference quartiles in women

Women	Neck circumference (cm)				*P*
	≤30	30–31	31–32	>32	
*N*	398	463	657	406	
Age (yr)	48.17 ± 13.97	49.81 ± 14.13	50.89 ± 13.78	52.14 ± 13.40	<0.01
weight (kg)	50.98 ± 4.66	53.81 ± 4.93	56.10 ± 5.28	56.87 ± 5.44	<0.01
Waist circumference (cm)	67.65 ± 6.20	70.94 ± 5.92	73.56 ± 6.18	76.32 ± 7.42	<0.01
Hip circumference (cm)	86.57 ± 4.48	88.60 ± 4.28	88.67 ± 4.75	91.57 ± 4.75	<0.01
Waist to hip ratio	0.78 ± 0.06	0.80 ± 0.07	0.81 ± 0.07	0.82 ± 0.08	<0.01
BMI (kg/m^2^)	21.11 ± 1.62	21.96 ± 1.66	22.54 ± 1.53	22.82 ± 1.68	<0.01
SBP (mmHg)	121.27 ± 19.90	121.87 ± 19.38	124.05 ± 20.09	128.69 ± 21.74	<0.01
DBP (mmHg)	72.53 ± 11.22	73.15 ± 11.52	74.24 ± 12.06	75.73 ± 12.75	<0.01
FBG (mmol/L)	5.30 ± 0.78	5.35 ± 1.05	5.46 ± 1.01	5.66 ± 1.26	<0.01
Fasting insulin (pmol/L)	27.5(20.5–37.5)	28.7(21.85–38.15)	33.3(25.0–47.2)	35.9(27.6–46.3)	<0.01
HbA1c (%)	5.10 ± 0.59	5.15 ± 0.69	5.21 ± 0.65	5.24 ± 0.95	<0.01
HOMA-IR	0.92(0.65–1.27)	0.97(0.69–1.32)	1.18(0.84–1.56)	1.25(0.88–1.80)	<0.01
Total cholerterol (mmol/L)	4.89 ± 0.96	5.00 ± 1.05	4.96 ± 1.03	4.98 ± 0.98	0.42
LDL-cholesterol (mmol/L)	2.75 ± 0.70	2.88 ± 0.72	2.84 ± 0.71	2.80 ± 0.68	0.03
HDL-cholesterol (mmol/L)	1.63 ± 0.31	1.59 ± 0.30	1.52 ± 0.30	1.46 ± 0.30	<0.01
Triglycerides (mmol/L)	1.02(0.75–1.36)	1.03(0.77–1.49)	1.11(0.84–1.56)	1.25(0.88–1.80)	<0.01
Creatinine (umol/L)	68.83 ± 9.63	69.50 ± 9.82	69.25 ± 10.68	67.25 ± 14.73	0.03
eGFR (ml/min/1.73^2^)	89.73 ± 15.36	88.17 ± 15.52	89.14 ± 16.64	92.181 ± 19.12	<0.05
ALT (U/L)	14(11–19)	14(11–19)	15(12–21)	16(13–22)	<0.01
AST (U/L)	21(18–25)	21(18–26)	21(18–26)	21(19–25)	0.17
NAFLD,%	3.02 %	5.39 %	8.52 %	12.56 %	<0.05

### Correlation coefficients for NC

After adjusting for age, partial correlation analysis demonstrated positive correlation between NC and BMI, WC, HC, systolic blood pressure, diastolic blood pressure, insulin, HOMA-IR, TG and ALT, regardless of sex. But NC level was negatively correlated with HDL-c among various metabolic features (Table 4). NC was positively related to fast blood glucose only in women while it was positively correlated to TC and LDL -c in male.

**Table 4:** Correlation between neck circumference and other parameters by sex

Variable	Men		Women	
	*r*	*p*	*r*	*p*
Weight (kg)	0.45	<0.01	0.36	<0.01
Waist circumference (cm)	0.40	<0.01	0.40	<0.01
Hip circumference (cm)	0.37	<0.01	0.33	<0.01
Waist to hip ratio	0.05	0.18	0.22	<0.01
BMI (kg/m^2^)	0.41	<0.01	0.30	<0.01
SBP (mmHg)	0.18	<0.01	0.08	<0.01
DBP (mmHg)	0.09	<0.01	0.06	<0.01
FBG (mmol/L)	0.06	0.09	0.10	<0.01
Fasting insulin (pmol/L)	0.11	<0.01	0.09	<0.01
HbA1c (%)	0.11	<0.01	0.06	<0.01
HOMA-IR	0.12	<0.01	0.17	<0.01
Total cholerterol (mmol/L)	0.10	<0.01	0.02	0.51
LDL-cholesterol (mmol/L)	0.16	<0.01	0.01	0.57
HDL-cholesterol (mmol/L)	−0.13	<0.01	−0.18	<0.01
Triglycerides (mmol/L)	0.14	<0.01	0.14	<0.01
Creatinine (umol/L)	0.11	<0.01	0.05	0.05
eGFR (ml/min/1.73^2^)	−0.13	<0.01	0.07	<0.01
ALT (U/L)	0.07	0.05	0.07	<0.01

All correlation coefficients were calculated after adjustment for age

### Relationship between NC and NAFLD

Using the binary logistical regression, the independent and significant clinical parameters associated with NAFLD were identified. The data analysis showed a strong positive association between HOMA-IR, BMI, TG and NAFLD, regardless of genders (all *P <*0.001). Among male participants, smoke status and NC levels were independently and significantly associated with NAFLD, even after adjusting for age, BMI, SBP, WC, TC, HOMA-IR, ALT and eGFR, the relationships were still existed. Using NC quartile 1 as a reference, the OR for NAFLD was 1.47 (95 % CI 0.79–2.75, *P <* 0.01), 2.07 (95 % CI 1.19–2.75, *p <*0.01), and 2.18 (95 % CI 1.16–4.13, *P <* 0.01) for quartiles 2, 3 and 4 respectively (Table 5). Additional adjustment for other potential confounding factors, ALT and WC were the independent and significant factors of NAFLD in female subjects.

**Table 5:** Adjusted ORs and 95 % CIs for NAFLD according to NC quartiles

	ORs (95 % CI)				*P* value for trend
NAFLD	Q11	Q2	Q3	Q4	
Model 1	1	1.76(0.97–3.23)	3.10(1.86–5.20)	3.98(2.21–7.16)	<0.01
Model 2	1	1.81(0.99–3.31)	3.04(1.81–5.12)	3.88(2.15–7.01)	<0.01
Model 3	1	1.58(0.85–2.92)	2.22(1.29–3.81)	2.36(1.27–4.40)	<0.01
Model 4	1	1.51(0.81–2.82)	2.22(1.29–3.63)	2.21(1.17–4.15)	<0.05
Model 5	1	1.47(0.79–2.75)	2.07(1.19–3.60)	2.18(1.16–4.13)	<0.05

Model 1 adjusted for age

Model 2 further adjusted for smoking

Model 3 further adjusted for BMI

Model 4 further adjusted for TG and HOMA-IR

Model 5 further adjusted for eGFR and ALT

## Discussion

In this cross-sectional analysis of non-obese Chinese population, prevalence of NAFLD was 10.94 % and the prevalence in males was obviously higher than in females (19.89 % vs 7.48 %). These results were comparable to previous studies conducted in non-obese individuals in Japan, Korea and China. The prevalence reported in these studies was 15.2 %, 12.6 % and 7.2 % respectively [7, 17, 18]. This may result from a difference in the study population, because all of those researches were performed in health checkups while we were community based cohort study. The gender difference for the prevalence of NAFLD could be mainly explained by the female hormones protection against NAFLD and less smokers in female population.

Furthermore, we showed that ALT, TG, NC levels and smoke status were independently and significantly associated with NAFLD in men. While in women, age, ALT, TG and HbA1c were independently and significantly associated with NAFLD. The roles of insulin resistance, alterations in lipid metabolism in NAFLD are well recognized. But here for the first time, we demonstrated that smoke status was a very important risk factor for developing NAFLD in non-obese males. While the mechanism for this association is unclear, several investigators have reported potential pathways. Hongwei et al. noted that cigarette smoking inactivates 5’- adenosine monophosphate-activated protein kinase (AMPK) by dephosphorylation and promoted triglyceride accumulation in hepatocytes via activation of sterol regulatory element binding protein-1 (SREBP-1), inducing fatty liver in mice fed a high fat diet [19]. In obese rats, cigarette smoking elevated ALT and caused hepatocellular ballooning and lobular inflammation [20]. Cigarette smoking is known to cause oxidative stress and oxidative stress is a known mechanism of injury in NAFLD [21–23]. A recent research reported that cigarette smoke exposure was associated with increased CYP2E1, which is linked to the mechanism of oxidative injury [24].

One interesting finding from our study was that NC, a proxy of upper-body sc fat, was a novel indicator for identifying NAFLD in normal weight males but not in females. NC is significantly associated with BMI, WC, HC, systolic blood pressure, diastolic blood pressure, insulin, HOMA-IR, triglycerides and ALT. NC is an alternative measurement for upper-body subcutaneous fat and is also a good indicator of ectopic fat distribution. Anatomically, upper-body subcutaneous fat is a unique fat depot located in a separate compartment compared with VAT. It has been demonstrated that upper-body sc fat is responsible for a much larger proportion of systemic free fatty acid release and this fat depot may play an important role in risk factor pathogenesis [11]. The liver plays a principal role in lipid metabolic pathways by taking up serum free fatty acid (FFA), and manufacturing, storing, and transporting lipid metabolites [25]. The accumulation of lipids in hepatocytes is the hallmark feature of the pathogenesis of NAFLD.

The differential predicting effect of NC by sex was controversy to previously literature. Previous analyses in the Framingham Heart Study have shown that fat depots are more strongly associated with an adverse risk factor profile in women compared with men [11]. One of the possible explanations for the different results in our study is the large proportion of smokers in males which may result in a greater free fatty acid delivery to the liver.

WC is often used as a surrogate marker of central obesity as well as markers of NAFLD. Despite the established use of the WC in the evaluation of health risk, it has a number of limitations [26]. First, different anatomical landmarks have been used to determine the exact location for measuring WC in different clinical studies [26]. The specific site used to measure the WC influences the absolute WC value that is obtained [26]. Second, it is subject to variations during the day and under health conditions affecting either the structure of the abdominal wall (e.g. severe obesity, lipoabdominoplasty, great weight loss) or abdominal organs and cavity [26]. Third, it may not be practical for large population studies, especially in cold weather and heavy clothing [26]. Measuring the NC is easier than measuring the WC, which presents a large variability in its procedure [26]. Therefore, NC may have many advantages over WC in screening NAFLD.

Obesity is a major risk factor for the development of NAFLD and even a small elevation in body weight will increase the risk of fatty liver formation [27, 28]. Meanwhile, previous literatures had showed that NC was an independent predictor for fatty liver disease in the general population. Our strong evidence indicating the correlation of NC and NAFLD even in normal weighted people may validate their results and provide valuable clues for further studies. We also indicated that smoke status and lipid deposition together may play important roles in the early stage of the development of NAFLD in male.

Despite the above findings of our project regarding the predicting role of NC in NAFLD, we had some limitations related to the cross-sectional experimental protocol. Even though the underlying mechanism of these gender-based differences could not have been clarified in this study, the gender dimorphism in the influence of regional fat on the risk of developing NAFLD may be an important area for further investigation [6].

## Conclusion

In summary, we demonstrated that NC is a useful and convenient tool for detecting NAFLD in non-obese men but not in women. The relationship with triglyceride supports the hypothesis that NC may be a good indicator of accumulation of lipid in the liver.
